# Death associated with coronavirus (COVID-19) infection in individuals with severe mental disorders in sweden during the early months of the outbreak

**DOI:** 10.1192/j.eurpsy.2021.339

**Published:** 2021-08-13

**Authors:** U. Werneke, M. Maripuu, M. Bendix, L. Öhlund, M. Widerstrom

**Affiliations:** 1 Sunderby Research Unit, Umeå University, Deparment of clinical science, division of psychiatry, Umeå, Sweden; 2 Department Of Clinical Sciences- Psychiatry, Umeå University, Umeå, Sweden; 3 Clinical Microbiology, Umeå University, Umeå, Sweden

**Keywords:** COVID-19, mental disorder, Mortality, coronavirus

## Abstract

**Introduction:**

Individuals with severe mental disorder (SMD) have a higher risk of somatic comorbidity and mortality than the rest of the population.

**Objectives:**

To assess whether individuals with SMD had a higher risk of death associated with a COVID-19 infection (COVID-19 associated death) than individuals without SMD.

**Methods:**

Exploratory analysis with a cross-sectional design in the framework of a population-based register study covering the entire Swedish population. The Swedish Board for Health and Welfare (Socialstyrelsen) provided anonymised tabulated summary data for further analysis. We compared numbers of COVID-19 associated death in individuals with SMD (cases) and without SMD (controls). We calculated the odds ratio (OR) for the whole sample and by age group and four potential risk factors, namely diabetes, cardiovascular disease, hypertension, chronic lung disease.

**Results:**

The sample comprised of 7,923,859 individuals, 103,999 with SMD and 7,819,860 controls. There were 130 (0.1%) COVID-19 associated deaths in the SMD group and 4945 (0.06%) in the control group, corresponding to an OR of 1.98 (CI 1.66-2.35; p < 0.001). The odds were fourfold in the age group between 60 and 79 years. Cardiovascular diseases increased the odds by 50%. Individuals with SMD without any of the risk factors under study had three-folds odds of COVID-19 associated death.

**Conclusions:**

Our preliminary results suggest that individuals with SMD are a further group at increased risk of COVID-19 associated death. The factors contributing to this increased mortality risk require clarification.

**Disclosure:**

Ursula Werneke has received funding for educational activities on behalf of Norrbotten Region (Masterclass Psychiatry Programme 2014-2018 and EAPM 2016, Luleå, Sweden): Astra Zeneca, Eli Lilly, Janssen, Novartis, Otsuka/Lundbeck, Servier, Shire and Sunovi

